# Exploring the genetic heterogeneity in major depression across diagnostic criteria

**DOI:** 10.1038/s41380-021-01231-w

**Published:** 2021-07-21

**Authors:** Bradley S. Jermy, Kylie P. Glanville, Jonathan R. I. Coleman, Cathryn M. Lewis, Evangelos Vassos

**Affiliations:** 1 Social, Genetic and Developmental Psychiatry Centre, Institute of Psychiatry, Psychology & Neuroscience, King’s College London, London, UK; 2 NIHR Maudsley Biomedical Research Centre, South London and Maudsley NHS Trust, London, UK; 3 Department of Medical & Molecular Genetics, Faculty of Life Sciences and Medicine, King’s College London, London, UK

**Keywords:** Genetics, Psychology, Depression

## Abstract

Major depressive disorder (MDD) is defined differently across genetic research studies and this may be a key source of heterogeneity. While previous literature highlights differences between minimal and strict phenotypes, the components contributing to this heterogeneity have not been identified. Using the cardinal symptoms (depressed mood/anhedonia) as a baseline, we build MDD phenotypes using five components—(1) five or more symptoms, (2) episode duration, (3) functional impairment, (4) episode persistence, and (5) episode recurrence—to determine the contributors to such heterogeneity. Thirty-two depression phenotypes which systematically incorporate different combinations of MDD components were created using the mental health questionnaire data within the UK Biobank. SNP-based heritabilities and genetic correlations with three previously defined major depression phenotypes were calculated (Psychiatric Genomics Consortium (PGC) defined depression, 23andMe self-reported depression and broad depression) and differences between estimates analysed. All phenotypes were heritable (h^2^_SNP_ range: 0.102–0.162) and showed substantial genetic correlations with other major depression phenotypes (Rg range: 0.651–0.895 (PGC); 0.652–0.837 (23andMe); 0.699–0.900 (broad depression)). The strongest effect on SNP-based heritability was from the requirement for five or more symptoms (1.4% average increase) and for a long episode duration (2.7% average decrease). No significant differences were noted between genetic correlations. While there is some variation, the two cardinal symptoms largely reflect the genetic aetiology of phenotypes incorporating more MDD components. These components may index severity, however, their impact on heterogeneity in genetic results is likely to be limited.

## Introduction

Major depressive disorder (MDD) is a common mental health condition characterised by periods of low mood [1, 2]. Heterogeneity within MDD is a key problem in research as it can mask associations with risk factors and restrict aetiological understanding [3]. Cai, Choi and Fried suggest heterogeneity arises in three ways. ‘Operationalisation’ relates to how the phenotype is defined and measured, ‘manifestation’ relates to the clinical presentation and ‘aetiology’ relates to a subgroups risk factor profile [4]. Heterogeneity research into the genetics of MDD has proven fruitful, highlighting, among other findings, variation in genetics of the individual symptoms [5] as well as differential polygenic risk profiles for atypical depression [6, 7], and early-onset MDD [8].

Operational heterogeneity has received particular attention recently due to the variation in phenotypes used in genome-wide association studies (GWAS) [3]. Major depression (MD)—a general definition of MDD in which cases may not have been defined using a structured clinical interview—has a significant heritable component, with estimates in the range of 20 and 50% [9–12]. It is a polygenic trait, meaning the genetic variance is explained partly by multiple common genetic variants of individually small effect in the population [13]. Due to its genetic architecture, large sample sizes of MD cases and controls are required to identify these variants [14]. Many GWAS have therefore used a pragmatic ‘minimal phenotyping’ approach where MD cases are identified according to a single, self-report, question [15–17]. Hyde et al. performed a GWAS on data from 23andMe taking the union of six self-report questions asking if the participant has ever been diagnosed with clinical depression [15]. Similarly, Howard et al. included a ‘broad depression’ phenotype derived in the UK Biobank from self-report responses to the question ‘Have you ever seen a general practitioner/psychiatrist for nerves, anxiety or depression?’ [16].

This approach has identified genetic variants associated with MD [18], however, the minimal phenotype approach has been criticised for its lack of specificity to MDD [19]. Using the UK Biobank, Cai et al. [19], analysed the impact of the minimal phenotyping approach compared to a strict definition of MD, derived from responses to the Composite International Diagnostic Interview-Short Form (CIDI-SF) [20]. The strict MD phenotypes showed higher SNP-based heritability than the minimal phenotypes, and the genetic correlations between them were below 1. This confirms that GWAS results depend on phenotype definition, but the study did not explore the richness of MDD phenotypes available beyond the case-control definition.

MDD has many components including (1) five or more of the nine depressive symptoms listed in DSM-5 (inclusive of at least one cardinal symptom, depressed mood or anhedonia), (2) functional impairment, (3) episode duration of at least 2 weeks, (4) persistence of depression during the episode and (5) episode recurrence. We note that episode recurrence is not required for diagnosis, but it is often used as an indicator of severity in research studies [9, 19, 21, 22].

In this study, we sought to understand how genetic aetiology varies when these components of MDD are included in a phenotype. For example, episode recurrence may index a phenotype with a stronger genetic contribution and may also capture a subset of genetic variants associated with recurrence. Previous literature investigating the role of each component suggests recurrence increases the twin heritability of MD, however, findings for functional impairment, episode duration and number of symptoms have been inconsistent [9, 23–26]. No studies have yet assessed how these findings translate to molecular studies and heritability estimates from genome-wide variants.

We used mental health data from the UK Biobank to define 32 depression phenotypes which systematically incorporate the five components. Through assessing patterns in SNP-based heritability and genetic correlations between the 32 depression phenotypes and the current European ancestries gold standard PGC MDD cohort, we aim to explore how the genetic aetiology of a MD phenotype varies in the presence of the five components. As a secondary aim, we repeated the genetic correlation analysis with two minimal phenotypes (23andMe self-reported and broad depression) to determine if these definitions show different patterns from the PGC MDD cohort, consistent with minimal phenotypes accounting for the MD components to differing degrees.

## Methods

### Data

The UK Biobank, a health study of 502,655 individuals, was used for this study [27]. We used responses to the CIDI-SF which formed part of the Mental Health Questionnaire (MHQ) to define our phenotypes [20, 28]. This voluntary web-based questionnaire was completed by 157 366 UK Biobank participants aged between 45 and 82 when completing the questionnaire.

### Characterisation of the phenotypes

The CIDI-SF contains questions relating to an individual’s worst episode of depression during their lifetime [20]. A phenotype which required either of the two cardinal symptoms (depressed mood/anhedonia) to be endorsed acted as a baseline definition of MD. Five components for MD, which build upon the cardinal symptoms, were defined from CIDI-SF questions, corresponding to: episode recurrence (Two or more depressive episodes in lifetime), the presence of five or more depressive symptoms, a long episode duration (episode > 6 months), the presence of functional impairment (affected life/activities either ‘somewhat’ or ‘a lot’) and the persistence of the depressive symptoms during the episode (felt depressed ‘almost every day’ or ‘every day’). For brevity, these components will be referred to as recurrence, symptoms, duration, impairment, and persistence, respectively. For each component, we derived a binary variable indicating if the individual endorsed this aspect of depression. For more detail as to how these binary variables were defined, please see Supplementary Table 1.

**Fig. 1:**
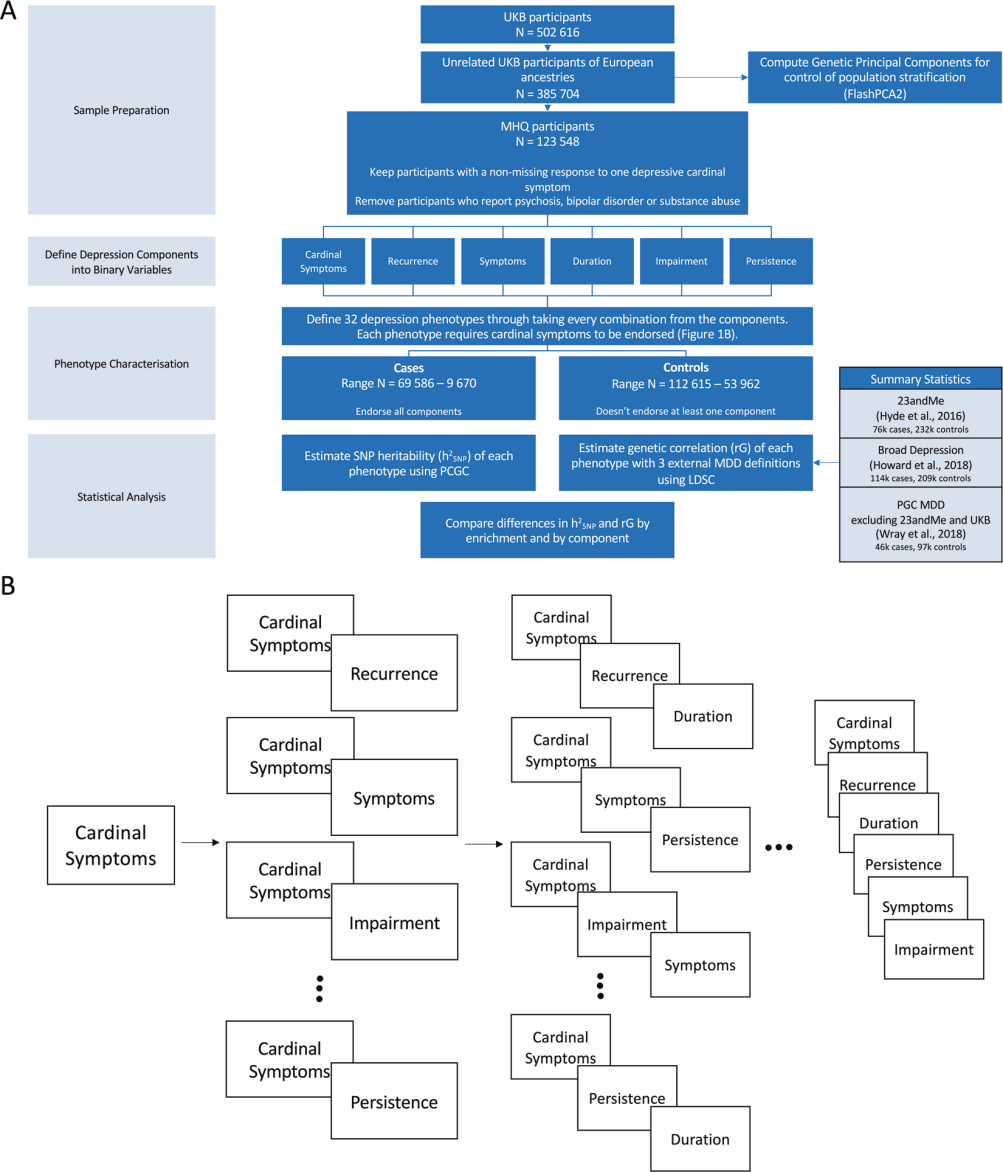
Workflow of the study design. **A** Flow chart of key methodological steps. UKB UK Biobank, PCGC phenotype correlation genotype correlation, LDSC linkage disequilibrium score regression, PGC psychiatric genomics consortium, MDD major depressive disorder. **B** Characterisation of the 32 phenotypes. **B** Provides a graphical image of each phenotypes composition. Each time a component is added an additional phenotype is defined. Taking all possible combinations from each addition creates a possible 32 distinct phenotypes. The ellipses have been included in the graph to represent the additional phenotypes not included within the figure.

In addition to the baseline phenotype which consists of all participants endorsing either of the two cardinal symptoms, phenotypes were created through taking every combination of each of the five binary components at the varying levels of enrichment. We use the term ‘enrichment’ to refer to the number of phenotypic components used to define a phenotype (between 1 and 5). This results in a total of 32 different phenotypes (Fig. 1).

The naming convention for each phenotype throughout the rest of the paper relates to which components are endorsed to be designated case status (Supplementary Table 2). For example, the phenotype ‘Cardinal + Recurrence + Impairment’ reflects all individuals who endorsed at least one cardinal symptom, report more than one major depressive episode and were at least somewhat functionally impaired during their worst episode.

Participants were designated as controls if one component within the phenotype was not endorsed. For example, where cardinal symptoms, recurrence and impairment are required for case status, if at least one of the three is not endorsed, the participant would be a control. Therefore, as a depression phenotype includes more components, the number of cases drops while the number of controls increases (Supplementary Table 3). For an evaluation of this approach to defining controls and its influence on the results, refer to the Supplementary Information. Controls were not ‘double-screened’ for the presence of any other psychiatric disorders, including MD, as to avoid upwardly biasing the SNP-based heritability and genetic correlation estimates [29–31]. However, participants were excluded independently of case/control status if they were identified as a possible case for schizophrenia, bipolar disorder or substance abuse (*N*_excluded_ = 3,032). This was determined through an individual self-reporting either the disorder or a relevant medication (Supplementary Table 4).

### Genetic data—quality control, SNP-based heritability and genetic correlations

#### Quality control

Participants in the final sample were unrelated and of European ancestries which were identified using a previously described analytical pipeline (Supplementary Methods) [27, 32, 33].

A total of 560,173 genotyped and 9,940,918 imputed SNPs remained after QC. Genotyped SNPs were used to estimate heritabilities and imputed SNPs were used to compute genetic correlations.

#### SNP-based Heritability

Phenotype-Correlation-Genotype-Correlation (PCGC; https://github.com/omerwe/S-PCGC) was used to estimate the SNP-based heritability of the 32 depression phenotypes [34, 35]. To convert to the liability scale, population prevalence was assumed to equal the sample prevalence prior to the application of any exclusion criteria for each phenotype (Supplementary Table 3). As recommended, the major histocompatibility complex region was removed (chromosome 6; 28,866,528–33,775,446 bp) reducing the total number of SNPs used to estimate the SNP-based heritability to 554,059 [36]. The first six genetic principal components, genotyping batch and assessment centre were included as covariates for all phenotypes.

#### Genetic correlation

Genetic correlations were computed using linkage disequilibrium score regression (LDSC) [37]. LDSC was chosen for this analysis as the summary statistics necessary for PCGC estimation were not available for the three external MD phenotypes and we did not have access to the individual level data to compute these. Correlations were estimated for each of the 32 phenotypes and PGC defined depression, using summary statistics from Wray et al. with 23andMe and UK Biobank samples removed (*N*_cases_ = 45,396, *N*_controls_ = 97,250) [17]. We repeated this analysis for broad depression, and 23andMe self-reported depression using summary statistics from Howard et al. (*N*_cases_ = 113,769, *N*_controls_ = 208,811) and Hyde et al. (*N*_cases_ = 75,607, *N*_controls_ = 231,747), respectively [15, 16]. The 32 depression phenotypes were residualised by the first six genetic principal components, genotyping batch and assessment centre, then a GWAS performed using PLINK 2.0 (cog-genomics.org/plink/2.0 [38, 39]) to obtain summary statistics for each phenotype. Pre-computed linkage disequilibrium scores, HapMap3 SNPs and the default settings of LDSC were used to calculate the genetic correlations for all phenotypes.

### Statistical analysis

#### How does enriching the major depression phenotype impact SNP-based heritability and genetic correlation?

We initially investigated the trend in SNP-based heritability and genetic correlations with enrichment of the MD phenotype to determine if the depth of information within a phenotype influences the genetic aetiology. Using the phenotype requiring only cardinal symptoms as a reference, we calculated differences in SNP-based heritability and genetic correlation estimates for the remaining 31 MD phenotypes. This was performed using a previously described block jackknife methodology with 200 blocks (Supplementary Methods) [40, 41]. The differences were then grouped according to the phenotypes level of enrichment, i.e. all differences for phenotypes requiring cardinal symptoms and one other component were grouped. Averages within these groups were then calculated by taking the inverse-variance weighted mean of the SNP-based heritability and genetic correlations (Supplementary Methods).

#### Component importance

We then investigated the relative effect each component had in driving any general pattern in the SNP-based heritability and genetic correlation estimates. For this test, differences in estimates are calculated using the same block jackknife approach. Differences were calculated between all combinations of phenotypes which differ by only one component. For example, the two phenotypes; ‘cardinal symptoms + recurrence’ and ‘cardinal symptoms + recurrence + symptoms’ would be compared as the phenotypes are identical other than for the symptoms component. Any difference in estimates is attributed to the component that differs between the two phenotypes (symptoms, in the example above). Each component may be added to multiple phenotypes with the same level of enrichment, for example, recurrence may be added to ‘cardinal symptoms + impairment’ and ‘cardinal symptoms + persistence’. To understand the average impact at this level of enrichment, the inverse variance weighted average difference was calculated.

## Results

### Phenotypes

From the 123,548 unrelated UKB participants of European ancestries who provided at least one non-missing response to the cardinal symptom items within the MHQ, 69,586 endorsed at least one cardinal symptom, and 9,670 of these endorsed all five components. Final sample sizes for all phenotypes are shown in Supplementary Table 3.

### SNP-based heritability

**Fig. 2:**
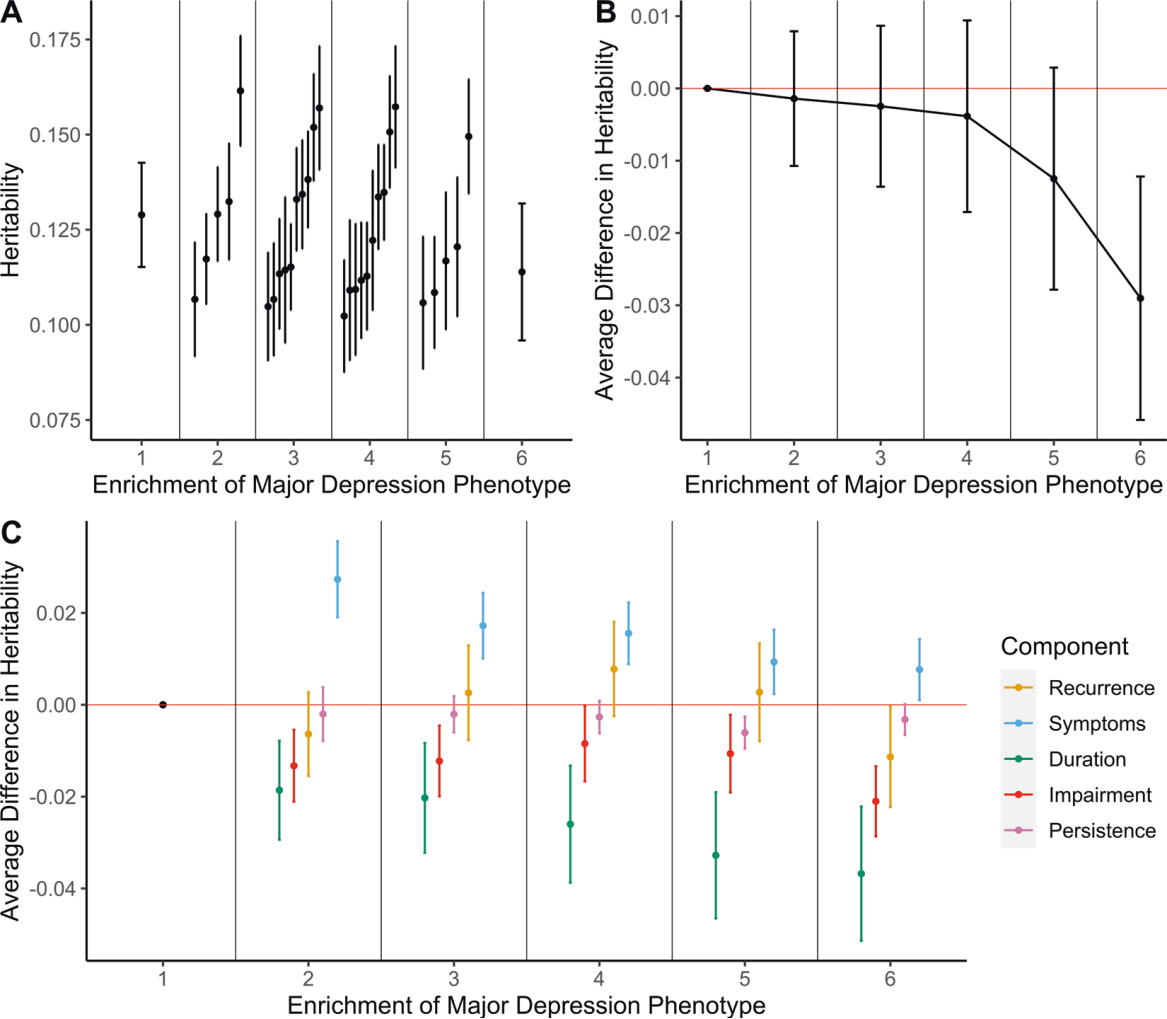
Analyses of SNP-based heritability of major depression phenotypes. **A** SNP-based heritability estimates on the liability scale for each phenotype grouped by phenotype enrichment. Phenotype enrichment is defined as the number of components considered to define case status. **B** Trend in SNP-based heritability estimates by phenotype enrichment. Each point estimate represents the average difference in SNP-based heritability relative to the phenotype which requires either of the cardinal symptoms to be endorsed. The cardinal symptom phenotype is the reference under the phenotype enrichment level 1. Error bars represent standard errors of the difference in SNP-based heritability estimates and averages were taken as the inverse-variance weighted mean of the enrichment group. **C** Trend in SNP-based heritability estimates by component. Each point estimate represents the average difference in SNP-based heritability induced from the addition of the component. Estimates are further grouped by level of phenotype enrichment. The point estimate with a phenotype enrichment of 1 is the cardinal symptoms phenotype and as such does not show any change due to the presence of no additional components. It is included for completeness. Errors bars represent standard errors of the difference in SNP-based heritability estimates and averages were taken as the inverse-variance weighted mean from all component comparisons within the enrichment group.

SNP-based heritability estimates for the 32 phenotypes ranged from 0.102 (SE = 0.015, Phenotype = Cardinal + Impairment + Persistence + Duration to 0.162 (SE = 0.014, Phenotype = Cardinal + Symptoms) (Fig. 2A). All estimates were significantly different from 0 following Bonferroni correction for multiple testing (*α*_Bonferroni_ < 0.0016 (0.05/32 phenotypes)) (Supplementary Table 5).

#### Trend with phenotypic enrichment

To understand the effect of enriching the MD phenotype, differences between the SNP-based heritability estimates relative to the phenotype of only cardinal symptoms were computed and averaged by the phenotypic enrichment, i.e. the number of components. As the phenotypes become more enriched, the average SNP-based heritability of the phenotype decreases (Fig. 2B). However, taking each level of enrichment in turn, none of the SNP-based heritabilities was significantly different from the SNP-based heritability of the phenotype with only cardinal symptoms (*p* > 0.05) (Supplementary Table 6).

#### Importance of each component

Analysis by enrichment averages all phenotypes by number of components, however, the components used to define the phenotypes within each enrichment group vary. As such, the averaging approach removes any specific effect of a component. To understand the impact of each component on SNP-based heritability, we calculated the change in SNP-based heritability after adding the component and took the average difference a component had on phenotypes with the same level of enrichment (see Supplementary Table 7 for every test performed as well as how the results were averaged).

The requirement of five or more symptoms during the episode induced a significant increase in SNP-based heritability when the component was added to the cardinal symptoms only phenotype after correcting for multiple testing (*α*_Bonferroni_ < 0.002 (25 tests = 5 components over 5 levels of enrichment)) (Difference in SNP-based heritability = 0.027; SE = 0.008; *p* value = 9.67 × 10^−4^). Inclusion of any components made no significant differences at all other levels of enrichment (Fig. 2C; Supplementary Table 7). The lack of significance limits comparisons across components; however, episode duration decreased SNP-based heritability to the greatest degree (on average 2.7% across all levels of enrichment). It is, therefore, likely this component is contributing greatest to the decrease in SNP-based heritability with increasing phenotype enrichment. Conversely, the presence of five symptoms increased SNP-based heritability to the greatest degree, with an average increase of 1.4% (Supplementary Table 7).

### Genetic correlation

**Fig. 3:**
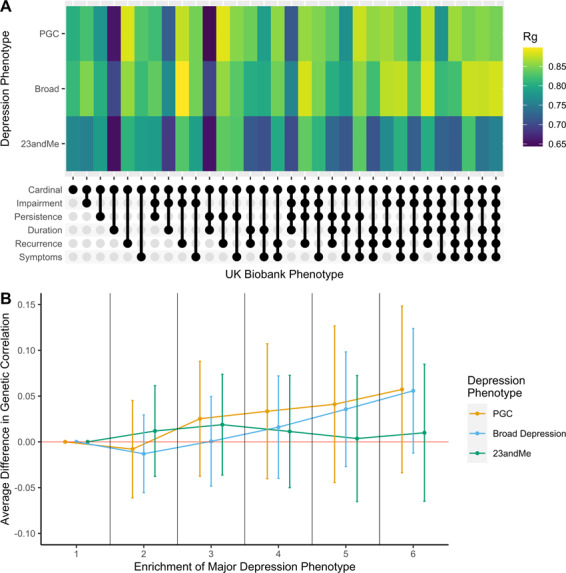
Analyses in genetic correlations with three previously defined major depression phenotypes. **A** A heatmap of genetic correlation estimates for each phenotype. Our defined phenotypes are displayed on the x-axis. The three previously defined major depression phenotypes are displayed on the y-axis. Note: the legend shows the scale for correlation estimate comparisons is between 0.65 and 0.85. **B** Trend in genetic correlation estimates with PGC defined major depression by phenotype enrichment. Point estimates show the average difference in genetic correlation relative to the cardinal symptom only phenotype. This is shown as the reference point under the first level of phenotype enrichment. Error bars represent the standard errors of the difference.

All genetic correlations were significantly different from 0 following Bonferroni correction for multiple testing (*α*_Bonferroni_ < 0.0016 (0.05/32 phenotypes)) (Supplementary Table 8). Genetic correlations ranged between 0.651–0.895 for PGC defined depression, 0.652–0.837 for 23andMe self-reported depression and 0.699–0.900 for broad depression (Fig. 3A).

#### Trend with phenotypic enrichment

Similar to the SNP-based heritability analyses, we analysed the effect of enriching the depression phenotype on the trend in genetic correlations with three external MD phenotypes. We compared all differences relative to the phenotype which required only cardinal symptoms to be endorsed. Differences in genetic correlation were not significant at any level of enrichment (*p* > 0.05) for broad depression, PGC or 23andMe defined depression. Both the broad depression and PGC defined depression showed an increase in genetic correlations estimates with enrichment of the depression phenotype, however, this is speculative and would require a study with greater power to show conclusively (Fig. 3B; Supplementary Table 9). 23andMe self-reported depression did not show such an increase by phenotypic enrichment (Fig. 3B; Supplementary Table 9)

#### Importance of each component

**Fig. 4:**
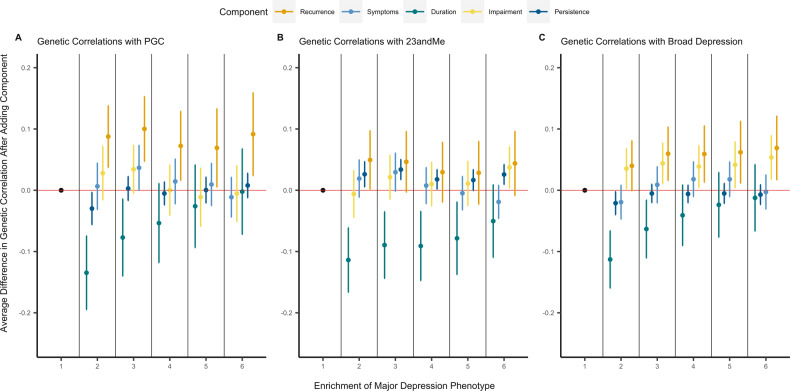
Trend in genetic correlation estimates by component. **A** Trend using PGC defined major depression phenotype as the comparison for genetic correlation computation. **B** Trend using 23andMe defined major depression phenotype as the comparison for genetic correlation computation. **C** Trend using broad depression phenotype as the comparison for genetic correlation computation. Each point estimate represents the average difference in genetic correlation induced from the addition of the component. Estimates are further grouped by level of phenotype enrichment. The point estimate with a phenotype enrichment of 1 is the cardinal symptoms only phenotype and as such does not show any change due to the presence of no additional components. It is included for completeness. Errors bars represent standard errors of the difference in genetic correlation estimates and averages were taken as the inverse-variance weighted mean from all component comparisons within the enrichment group.

We analysed the change in genetic correlation induced by the addition of MD components for the three MD phenotypes. The addition of duration to the phenotype requiring only cardinal symptoms decreased the genetic correlation with all three of the MD phenotypes at a level of nominal significance (PGC: Δrg = −0.135; 23andMe: Δrg = −0.114; broad depression: Δrg = −0.113; *p* < 0.05). Similarly, the addition of persistence to phenotypes that consisted of cardinal symptoms and one other component on average increased the genetic correlation with the 23andMe phenotype (23andMe: Δrg = 0.034, *p* < 0.05. However, none of these associations survived correction for multiple testing (*α*_Bonferroni_ < 0.002 (0.05/25)) (Fig. 4; Supplementary Table 10).

Given this lack of association it is difficult to discern any real trend by component, however, incorporating recurrence into the depression phenotype resulted in the greatest average increase over all three of the depression phenotypes (average change: PGC = 8.8%; 23andMe = 4%; Broad depression = 6.4%). Conversely, incorporating episode duration into the depression phenotype consistently decreased the genetic correlation for all three depression phenotypes (average change: PGC = −5.1%; 23andMe = −8.5%; Broad depression = −4.4%).

## Discussion

In this study, we aimed to determine how the genetic aetiology varies by definition of MD. By defining five depression components in addition to the cardinal symptoms, we compared how the SNP-based heritability and genetic correlations with three previous depression studies varied with the presence and absence of each component.

Variability in SNP-based heritability across the phenotypes was low with a range of 5.9%. Relative to the cardinal symptom phenotype, the greatest increase in SNP-based heritability was 2.7%. Conversely, the greatest decrease was 3.9%. We caveat the latter with the fact that this difference is not statistically significant. This lack of variability suggests the genetic aetiology of the cardinal symptoms phenotype largely reflects that of other phenotypes which include more components of MD.

The phenotype with the highest SNP-based heritability was also parsimonious, requiring a cardinal symptom, and five or more symptoms during the episode. The increase in SNP-based heritability, from 12.9% for cardinal symptoms to 16.2% for cardinal symptoms and five or more symptoms was significant, which suggests the inclusion of five or more symptoms as a component leads to a more heritable phenotype. The parsimonious nature of this phenotype, and the fact that richer phenotypes had lower SNP-based heritability, closer to that of the cardinal symptoms, is an important finding as it indicates much of the differentiable heritable signal for MD over and above the cardinal symptoms may be captured from a careful assessment of symptoms of depression. Thorp et al. [5] show SNP-based heritability varies between 6 and 9% for individual symptoms of MD. It is therefore plausible that further heterogeneity for SNP-based heritability within this component exists and that certain symptom profiles will be more heritable than others. In practice, accurately estimating SNP-based heritability depends on selecting an accurate population prevalence to convert to the liability scale [42]. If this is mis-estimated, differences in estimates will arise artificially. All else being equal, the difference in SNP-based heritability between the cardinal symptoms and ‘Cardinal + Symptom’ phenotypes would be reduced if we either under-estimated the cardinal symptom or over-estimated ‘Cardinal+Symptom’ population prevalence.

While episode duration of greater than 6 months did not survive correction for multiple testing, the point estimates showed an effect of decreasing SNP-based heritability when incorporated into the phenotype. Indeed, a phenotype requiring the cardinal symptoms and a long episode duration is consistent with the depressive condition of dysthymia, which is considered a distinct diagnosis relative to more episodic depressive episodes in the DSM-5 [1]. This finding is corroborated by a twin study which showed a negative relationship between monozygotic-dizygotic concordance ratio and episode duration [23]. While this could suggest a phenotype closer to that of dysthymia is less heritable, measurement error in the retrospective assessment of episode duration can introduce “noise” into the phenotype, reducing SNP-based heritability. Indeed, test-retest reliability for episode duration has been shown to be low [43] and it is not a predictor of a concordant diagnosis at follow-up unlike other components, such as impairment or number of symptoms [44]. More generally, varying degrees of measurement error across items could play a role in the differences between SNP-based heritabilities and genetic correlations. Indeed, it has been shown a more reliable diagnosis of MD leads to increased heritability [45].

Recurrence has consistently been shown to increase the heritability of MD which we were unable to corroborate [9, 11, 12, 19, 22]. Given Cai et al. [19] show recurrence increases heritability using the same data and defined two phenotypes with similar definitions to ours in the UK Biobank, it offered us the opportunity to explore methodological reasons behind this lack of replication (See Supplementary Information for results and in-depth discussion). SNP-based heritabilities for LifetimeMDD and MDDRecur were higher in Cai et al. [19] by 12.5% (13.8% vs. 26.3%) and 17% (15.1% vs. 32.1%), respectively. Our supplementary analysis shows these can be attributed to two differences in our phenotypes: definitions of controls, and in the threshold for functional impairment applied. For controls, we included participants with sub-threshold levels of impairment as well as single-episode cases when defining recurrent depression to reduce the risk of a discontinuity when transforming to the liability scale and inflating SNP-based heritability estimates [29, 31]. Removing these participants reduces the difference to 8.1 and 8.9% for LifetimeMDD and MDDRecur. Further, Cai et al. [19] used a more severe threshold for functional impairment. Including this threshold, and therefore also removing participants who were ‘somewhat’ impaired, accounts for the remainder of the difference in SNP-based heritabilities. We recognise our control definition increases the risk of bias due to misclassification [46], however, we believe our definition finds a good balance between the two potential biases (see Supplementary Information for a more detailed discussion). More broadly, this comparison highlights the importance of the control group definition in SNP-based heritability estimates and the appropriate use of liability scale conversions [29, 31]. In contrast to some of the previous literature, we used retrospective self-report to define lifetime cases which has been shown to have recall bias [47, 48]. Prospective assessment may produce different results.

The genetic correlations between the cardinal symptom only phenotype and the three depression phenotypes were high (PGC: rg = 0.807, SE = 0.054; 23andMe: rg = 0.762, SE = 0.041; Broad depression: rg = 0.815, SE = 0.032). Recurrence and duration were the two components to increase and decrease the genetic correlation point estimates, showing a consistent pattern across the three MD definitions. However, while there was variation around the correlation estimate given by the cardinal symptom phenotype (PGC: Range = −15.6–8.8%; 23andMe: Range = −11.0–7.5%; Broad depression: Range = −11.6–8.4%), we found no evidence for a statistically significant increase or decrease either by phenotype enrichment or by component. As such, we cannot conclude that MD components change the specific set of associated genetic variants. The relative increase that can be induced from the components is limited due to the high correlation between MD and the cardinal symptom only phenotype. As such, a ceiling effect is imposed which would require large sample sizes to detect significant differences for such correlations.

In line with the findings from Cai et al. [19], we previously hypothesised the two minimal phenotypes (broad depression in UK Biobank and self-reported depression in 23andMe) are composed of a case sample with greater heterogeneity. Given the potential for inclusion of milder cases, we expected the minimal phenotypes to correlate with milder depression phenotypes to a greater extent relative to more enriched depression phenotypes. In contrast, we hypothesised PGC defined depression, being our gold standard, would show a positive trend between phenotypic enrichment and genetic correlation. No conclusive trend could be found by phenotype enrichment in any case suggesting differences are likely to be difficult to find given the large sample size employed within this study.

## Limitations

The results from this study should be evaluated in the context of the following limitations. To accurately estimate and compare heritabilities, the prevalence of the phenotype within the population must be estimated accurately. No previous literature exists for most of our phenotypes, so an assumption was made that UK Biobank represents a random subset of the population. This assumption is strong, given participants of the UK Biobank have been shown to have a higher socio-economic status and lower mortality rates than the rest of the UK [49]. The subset of participants who responded to the MHQ also has a lower rate of mental health-related hospital diagnoses [50]. Future consideration towards developing a representative dataset free from selection bias would help improve the validity of the prevalence’s used in this study.

We assume the difference in SNP-based heritability and genetic correlation estimates is attributable to the component that has been changed between the two phenotypes. It is likely in practice that this component covaries with risk factors for depression and even other components, such as depression. For example, should you endorse the five symptoms of depression you are also more likely to endorse recurrence. This limitation may be unpicked through investigating the set of participants who endorse one component but not the other, i.e. those that endorse recurrence but not the five symptoms, however, much greater sample sizes are required for such an analysis and the translational interpretation is less clear.

With respect to the genetic correlation analysis, we considered the PGC defined phenotype to be the gold standard for comparison against minimal phenotypes. It is indeed the case that this phenotype is the most stringently assessed for individuals of European ancestries, however, given MD’s inherent heterogeneity, it is unlikely all cases within this phenotype are recurrent or have had episodes of long duration. An equivalent external phenotype in which all components were known to be endorsed for all cases would be able to show more conclusively if the incorporation of the component provides more genetically comparable phenotypes. However, this is not how MD is currently defined in the diagnostic criterion so while more severe MD phenotypes [51–53] may behave as a better positive control for this study, it would only reflect a small subset of the total MD cases in the population.

## Conclusion

Despite an 86% reduction in cases between our least and most strict definitions of MD, we show comparatively low variability in SNP-based heritability and genetic correlations, suggesting diagnostic components do not play a key role in the heterogeneity of genetic results. We find evidence that out of the additional criteria typically used to establish diagnosis or severity of depression, incorporating five or more symptoms into the phenotype produces a significant increase in SNP-based heritability. While these components may be used to reduce misclassification between controls and cases and enhance power in GWAS, they do not appear key to identifying any distinct genetic aetiology of MD.

## Supplementary information

Supplementary Information

Supplementary Table 1

Supplementary Table 2

Supplementary Table 3

Supplementary Table 4

Supplementary Table 5

Supplementary Table 6

Supplementary Table 7

Supplementary Table 8

Supplementary Table 9

Supplementary Table 10

Supplementary Table 11

Supplementary Table 12

## Data Availability

The full GWAS summary statistics for the 23andMe discovery dataset will be made available through 23andMe to qualified researchers under an agreement with 23andMe that protects the privacy of the 23andMe participants. Please visit https://research.23andme.com/collaborate/#dataset-access for more information and to apply to access the data. All other GWAS summary statistics are publicly available from the following websites https://www.med.unc.edu/pgc/download-results/ (PGC summary statistics) and https://datashare.is.ed.ac.uk/handle/10283/3083 (broad depression summary statistics).

## Code availability

Code used for this study is available at https://github.com/Bjermy/decomposingMDD.
